# Outer retina dysfunction and choriocapillaris impairment in type 1 diabetes

**DOI:** 10.1038/s41598-021-94580-z

**Published:** 2021-07-26

**Authors:** M. Parravano, L. Ziccardi, E. Borrelli, E. Costanzo, S. Frontoni, F. Picconi, V. Parisi, R. Sacconi, A. Di Renzo, M. Varano, G. Querques

**Affiliations:** 1 IRCCS - Fondazione Bietti, Rome, Italy; 2 Department of Ophthalmology, IRCCS Ospedale San Raffaele, University Vita-Salute, Via Olgettina, 60, Milan, Italy; 3 Unit of Endocrinology, Diabetes and Metabolism, S. Giovanni Calibita Fatebenefratelli Hospital, Department of Systems Medicine, University of Rome Tor Vergata, Rome, Italy

**Keywords:** Retinal diseases, Type 1 diabetes

## Abstract

To study the outer retina morpho-functional characteristics and the choriocapillaris (CC) features in type 1 diabetic (T1D) patients, with and without signs of diabetic retinopathy (NPDR and NoDR). Twenty-five NPDR and 18 NoDR eyes were imaged by Optical Coherence Tomography Angiography. Ellipsoid zone (EZ) “normalized” reflectivity and CC perfusion density parameters, as flow deficits number (FDn), flow deficit average area (FDa) and flow deficit percentage (FD%), were analysed. Multifocal electroretinogram (mfERG) response amplitude densities (RADs) were measured. Mean EZ “normalized” reflectivity, CC FDn and FD% values, were similar (*p* > 0.05) in both groups, FDa was significant greater (*p* > 0.05) in NPDR compared with NoDR eyes. MfERG-RADs were similar in both groups. NPDR eyes showed a significant (*p* < 0.05) linear correlation between RADs and both, CC FDa and FD%. The EZ “normalized” reflectivity was negatively correlated with CC FD% in NoDR eyes. In NPDR T1D eyes a significant relationship between abnormal outer retina functional responses and CC impairment was observed, while in NoDR eyes the photoreceptor reflectivity was correlated to CC abnormalities. The outer retina dysfunction in NPDR correlated to CC drop-out let hypothesize that the outer retinal elements are functionally impaired in proportion to the CC vascular supply deficit.

## Introduction

Type 1 diabetes (T1D) is an autoimmune disorder that leads to a multi-organ microvascular impairment, with a prevalence of diabetic retinopathy development of 17% for diabetes duration < 5 years, reaching the 98% for 15 years or more of systemic disease^1,2^.

Several studies showed that these microvascular changes can affect both retinal and choroidal vasculature. By using the optical coherence tomography angiography (OCTA) it was possible to detect the non-perfusion areas at different retinal layers, in particular at the level of deep capillary plexus (DCP) that nourishes the middle and outer retina^3–6^.

More recently, the choriocapillaris (CC) and choroid (Ch) have been recognized by means of optical coherence tomography (OCT)/OCTA as key-players in the diabetic pathological mechanism, and the name of “diabetic choroidopathy” was coined to describe the CC hypoperfusion as a relevant and early physio-pathological process in diabetic patients^3^.

The CC and Ch sustain the high metabolic rate of the outer retinal layers and retinal pigment epithelium, contributing to the photoreceptor oxygen supply^7^. The photoreceptor structural integrity can be quantitatively investigated measuring the ellipsoid zone (EZ) reflectivity, a novel standardized methodology that was used to quantify photoreceptors’ damage in different disorders, including type 2 diabetic retinopathy (DR)^3,8^.

In T1D patients, multifocal electroretinogram (mfERG) abnormalities have been reported^9,10^, supporting the concept of early neuro-retinal dysfunction before retinal vascular signs of diabetic retinopathy are visible. However, due to the novel concept of diabetic choroidopathy^3^, it would be of a great interest to understand whether there exists an influence of CC and Ch changes on outer retinal function in T1D patients.

Therefore, the aim of our study was to study the outer retina morpho-functional characteristics and the choriocapillaris features in T1D patients with and without signs of diabetic retinopathy, by using OCTA and mfERG, for investigating on the potential influence of CC and Ch changes on outer retinal function.

## Results

Forty-three eyes of 43 T1D patients (22 females and 21 males) were included; 25 with signs of non-proliferative diabetic retinopathy (NPDR group) (11 females and 14 males) and 18 without signs of diabetic retinopathy (NoDR group) (11 females and 7 males). Of the 25 NPDR group, 14 eyes had mild and 11 moderate NPDR. Demographic results were showed in Table 1. The mean ± SD best corrected visual acuity (BCVA) was 86 ± 7.63 Early Treatment Diabetic Retinopathy Study (ETDRS) letters (range 20/12.5 to 20/40 Snellen) in NPDR group and 86.35 ± 3.98 ETDRS letters (range 20/16 to 20/25 Snellen) in NoDR group (*p* = 0.064).

**Table 1:** Demographics of participants: diabetic patients without diabetic retinopathy (NoDR) and with mild signs of diabetic retinopathy (NPDR).

	Number (n)	Gender (M/F)	Eyes (n)	Age (years)	Diabetes duration (years)	HbA1c (%)
NoDR	18	7/11	18	36.56 ± 13.29 19–66	16.22 ± 9.35 5–35	7.69 ± 0.51
NPDR	25	14/11	25	44.12 ± 11.22 23–68	21.04 ± 12.31 3–47	7.52 ± 1.08

### Enface OCTA analysis

No statistically significant differences have been found between NoDR and NPDR groups in EZ “normalized” reflectivity, CC Flow Deficit number (FDn), CC Flow Deficit percentage (FD%).

The mean ± SD CC Flow Deficit average area (FDa) was statistically significant greater in NPDR group (37.12 ± 14.17) compared with NoDR group (27.82 ± 7.55) (*p* = 0.018) (Table 2).

**Table 2:** Descriptive (mean ± SD) and inferential statistics (F;p) for OCT/OCTA values in diabetic patients without diabetic retinopathy (NoDR) and mild signs of diabetic retinopathy (NPDR).

	NPDR (mean ± SD)	NoDR (mean ± SD)	NoDR vs NPDR (F; p)
EZ “normalized” reflectivity	0.85 ± 0.12	0.86 ± 0.09	0.11; 0.746
CC FDn	5445.9 ± 475.1	6122 ± 1629	3.60; 0.065
CC FDa	37.12 ± 14.17	27.82 ± 7.55	**6.11; 0.018**
CC FD%	19.60 ± 5.89	16.67 ± 5.11	2.78; 0.103

EZ = ellipsoid zone, CC = choriocapillaris, FDn = flow deficit number, FDa = flow deficit average area, FD% = flow deficit percentage.

The bold type highlights the statistically significant p-values (p< 0.05).

**Figure 1:**
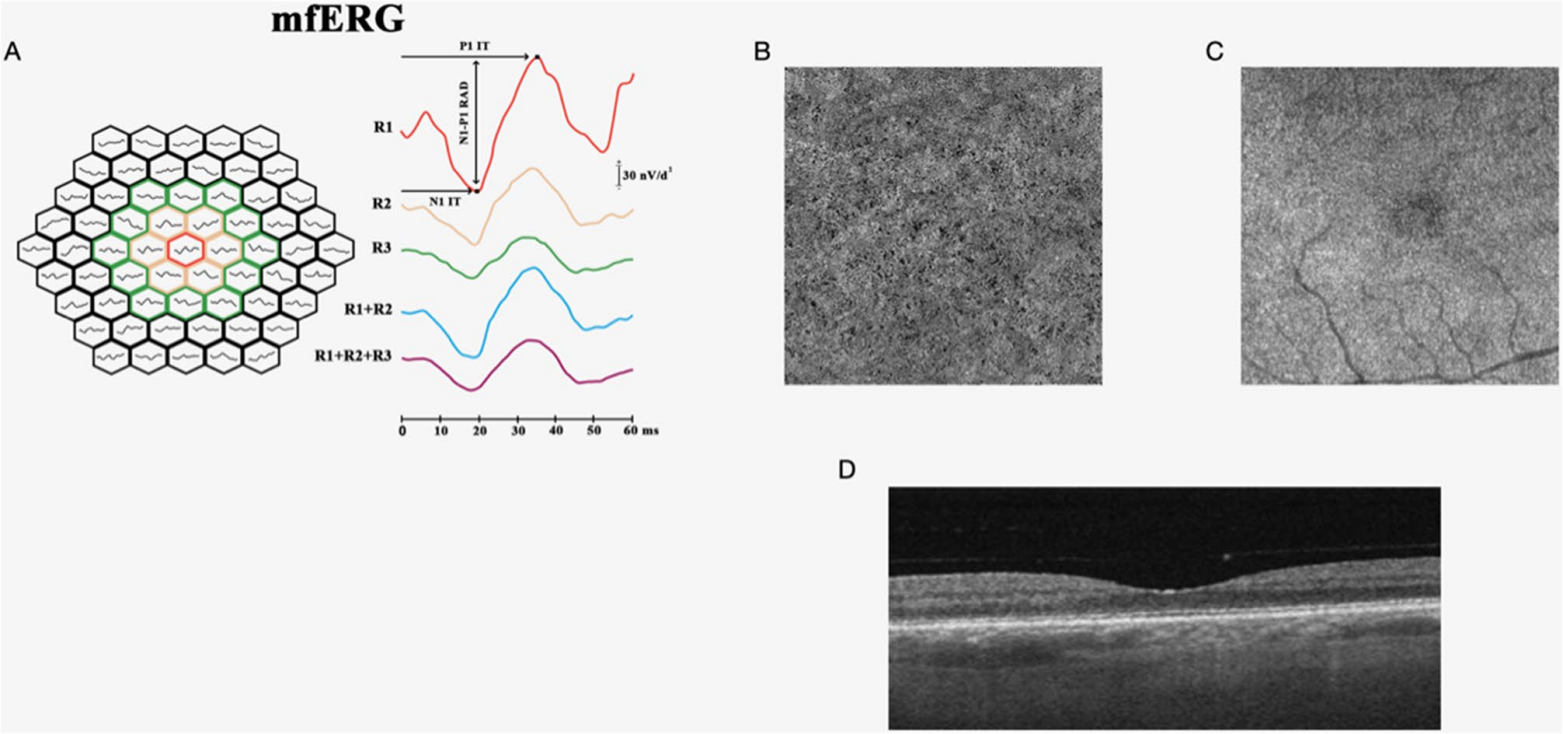
Example of images from one of our patients. (**A**) Multifocal electroretinogram (mfERG) bioelectrical responses in one representative eye affected by non-proliferative diabetic retinopathy (NPDR). The 61 hexagons trace arrays, subtending 15° of visual field, were sub-grouped in concentric annular areas (rings, R). We measured the N1-P1 response amplitude density (RAD) of mfERG responses recorded from three isolated and combined averaged rings with increasing eccentricity from the fovea: 0 to 2.5 (R1), 2.5 to 5 (R2), 5 to 10 (R3), 0 to 5 (R1 + R2), 0 to 15 (R1 + R2 + R3) degrees. (**B**) Choriocapillaris optical coherence tomography angiography (OCTA) slab acquired with PLEX Elite device. Several small regions of signal voids (dark spots scattered throughout the image) are evident on the enface OCTA image of the choriocapillaris and represent regions of decreased perfusion. (**C**) Ellipsoid zone (EZ) OCTA slab acquired with PLEX Elite device. The structural image at the level of the EZ shows regions of reduced reflectivity, which constitutes a surrogate for photoreceptor damage. (**D**) Corresponding structural optical coherence tomography acquired with PLEX Elite device.

Figure 1 shows the OCTA features in NPDR group.

### Correlation analysis

The EZ “normalized” reflectivity was negatively correlated with CC FD% in NoDR (*p* = 0.001), while no correlation was found in NPDR group. All the other relationship showed no statistically significance.

We correlated the EZ “normalized” reflectivity, the CC FDn, the CC FDa and the FD% with age, diabetes duration and glycated hemoglobin (HbA1c) levels. In NPDR group we found a significant relationship between age and CC FDa (*p* = 0.02) and FD% (*p* = 0.007), respectively; no significant correlation was found between age and both, EZ “normalized” reflectivity and FDn. In NPDR group the diabetes duration and HbA1c were not correlated with EZ “normalized” reflectivity (*p* = 0.824 and *p* = 0.179, respectively), CC FDn (*p* = 0.949 and *p* = 0.532, respectively), CC FDa (*p* = 0.128 and *p* = 0.433, respectively) and FD% (*p* = 0.129 and *p* = 0.621, respectively).

In NoDR group the age was significantly correlated with CC FDa (*p* = 0.011), while no other correlation was found with EZ “normalized” reflectivity (*p* = 0.135), FDn (*p* = 0.377) and FD% (*p* = 0.088); the diabetes duration was significantly correlated with both, CC FDa (*p* = 0.009) and FD% (*p* = 0.027), while no correlation was found with EZ “normalized” reflectivity (*p* = 0.082) and FDn (*p* = 0.637); none of these parameters was correlated with HbA1c levels (*p* = 0.860 for EZ “normalized” reflectivity, *p* = 0.438 for CC FDn, *p* = 0.524 for CC FDa, *p* = 0.319 for CC FD%).

### MfERG data

When comparing mean values of mfERG response amplitude density (RAD) recorded from isolated rings (R1, R2, R3) and combined rings (R1 + R2 and R1 + R2 + R3), not statistically significant (*p* > 0.05) differences were found between NoDR and NPDR eyes (Table 3).

**Table 3:** Descriptive (mean ± SD) and inferential statistics (one-way analysis of variance, ANOVA) for multifocal electroretinogram N1-P1 response amplitude density (RAD) values in diabetic patients without diabetic retinopathy (NoDR) and mild signs of diabetic retinopathy (NPDR).

	NoDR (nV/deg^2^)	NPDR (nV/deg^2^)	NoDR vs NPDR (F; p)
R1	90.34 ± 33.21	84.43 ± 21.38	0.50; 0.482
R2	33.54 ± 9.23	32.86 ± 8.24	0.07; 0.800
R3	19.67 ± 6.05	19.38 ± 3.96	0.03; 0.854
R1 + R2	35.11 ± 10.78	34.74 ± 9.58	0.01; 0.908
R1 + R2 + R3	22.61 ± 6.59	22.66 ± 4.86	0.08; 0.772

R1 = ring 1, 0°–2.5° foveal eccentricity; R2 = ring 2, 2.5°–5° foveal eccentricity; R3 = ring 3, 5°°–10° foveal eccentricity; R1 + R2 = area covering 0°–5° foveal eccentricity; R1 + R2 + R3 = area covering 0°–10° foveal eccentricity.

We explored the relationship between mfERG data with structural CC findings in both T1D groups.

**Figure 2:**
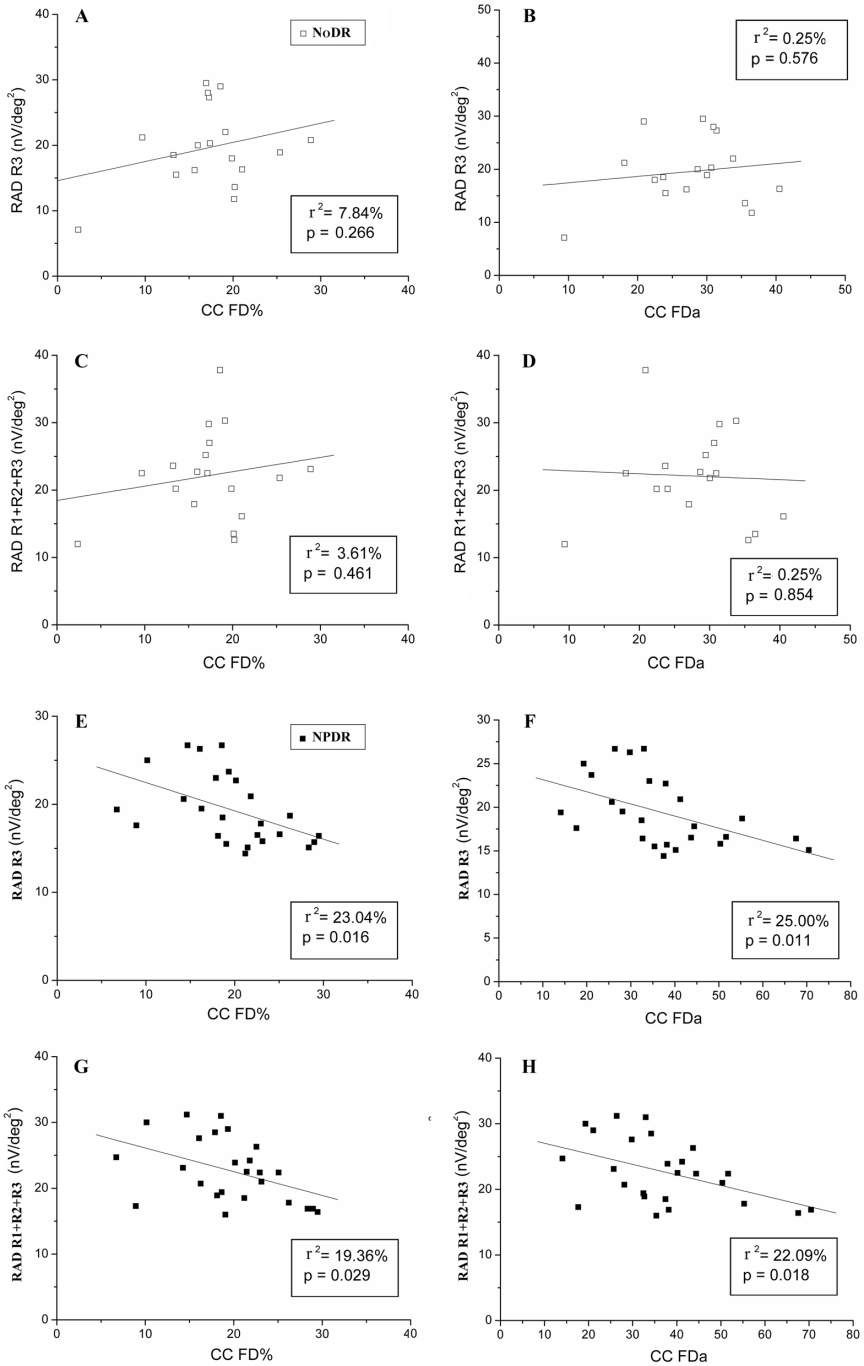
Scatter plots showing the linear correlations between the multifocal response amplitude density (RAD) derived from signals collected in an annular area enclosed between 5° and 10° (Ring 3, R3) or in the wider area between 0° and 10° (R1 + R2 + R3) of foveal eccentricity and the OCTA parameters of choriocapillaris flow deficit percentage (CC FD%) or choriocapillaris flow deficit area (CC FDa) in NoDR and NPDR eyes.

We found no significant linear (*p* > 0.05) correlations between mean RADs and choriocapillaris features (CC FDn, CC FDa and CC FD%) in NoDR group. By contrast, NPDR eyes showed a significant (*p* < 0.05) linear correlation between mean RADs from R3 and R1 + R2 + R3 and both CC FDa and CC FD% from almost superimposable areas (Table 4 and Fig. 2). This level of significance of the correlation between functional and morphological data was found in the same areas also when the two NoDR and NPDR groups were pooled together (data not shown). In the correlation between mean values of RADs and CC FDn from R3, we found a trend of statistical significance (*p* = 0.075). No other significant correlations between RADs and CC characteristics were found in other examined areas.

**Table 4:** Pearson’s linear correlations (R^2^;p) between optical coherence tomography angiography (OCTA) and multifocal electroretinogram N1-P1 response amplitude density (RAD) values in diabetic patients without diabetic retinopathy (NoDR) and with mild signs of diabetic retinopathy (NPDR).

	NoDR				NPDR			
	EZ “normalized” reflectivity	CC FDn	CC FDa	CC FD%	EZ “normalized” reflectivity	CC FDn	CC FDa	CC FD%
R1 RAD	0.64%; 0.745	2.56%; 0.522	14.44%; 0.134	19.36%; 0.067	4.41%; 0.307	6.76%; 0.751	0.36%; 0.762	0.00; 0.977
R2 RAD	5.29%; 0.357	3.61%; 0.399	0.36%; 0.822	3.61%; 0.451	0.81%; 0.671	4.00%; 0.360	10.89%; 0.104	11.56%; 0.095
R3 RAD	0.16%; 0.882	14.44%; 0.118	0.25%; 0.576	7.84%; 0.266	0.36%; 0.772	14.40%; 0.075	**25.00%; 0.011**	**23.04%; 0.016**
R1 + R2 N1-P1 RAD	0.81%; 0.721	10.24%; 0.190	0.25%; 0.859	2.89%; 0.510	0.01%; 0.967	5.76%; 0.267	9.00%; 0.139	7.84%; 0.175
R1 + R2 + R3 N1-P1 RAD	0.36%; 0.826	19.36%; 0.070	0.25%; 0.853	3.61%; 0.461	2.89%; 0.931	12.25%; 0.104	**22.09%; 0.018**	**19.36%; 0.029**

R1 = ring 1, 0°–2.5° foveal eccentricity; R2 = ring 2, 2.5°–5° foveal eccentricity; R3 = ring 3, 5°–10° foveal eccentricity; R1 + R2 = area covering 0–5° foveal eccentricity; R1 + R2 + R3 = area covering 0°–10° foveal eccentricity; EZ = ellipsoid zone, CC = choriocapillaris, FDn = flow deficit number, FDa = flow deficit average area, FD% = flow deficit percentage.

The bold type highlights the statistically significant p-values (p< 0.05).

Mean mfERG RADs from all rings plotted as a function of age, diabetes duration and HbA1c level were not significantly linearly correlated in NoDR group (all *p* > 0.05).

In NPDR group we found a significant linear correlation between age and mfERG RADs from R2, R3 and R1 + R2 (*p* = 0.047, *p* = 0.010 and *p* = 0.05 respectively). In these relationships, the correlation line had a negative slope thus meaning that reduced parafoveal RADs were correlated with increasing age in NPDR eyes. No other significant correlations between electrophysiological data and diabetes duration and HbA1c% were detected.

## Discussion

In this study we explored the morpho-functional features of outer retina and the CC and Ch in patients with T1D with and without signs of diabetic retinopathy.

To do so, we employed OCT/OCTA analysis and mfERG recordings. Overall, we found a negative correlation between EZ “normalized” reflectivity and CC features (FD%) in T1D eyes without DR. More importantly, we showed a significant relationship between abnormal outer retina functional responses and CC features (CC FDa and CC FD%) in NPDR T1D eyes (Fig. 1). For the best of our research, we excluded patients affected with macular edema.

The novel approach for quantification of photoreceptors’ impairment, as for as the EZ “normalized” reflectivity^3,8^, allowed to solve the obstacles caused by restricted lateral resolution and the OCT structural brightness limitations that could affect the standard photoreceptors’ analysis. As specified above, this recently reported technique was employed to describe damage of the photoreceptors in NPDR eyes as compared to controls, providing for the first time a quantitative evidence of photoreceptor damage in diabetic patients with signs of diabetic retinopathy^3^.

In our study on T1D patients no significant differences in EZ “normalized” reflectivity between NoDR and NPDR groups were observed. This parameter that could be used as surrogate of photoreceptor impairment seems to be not influenced by the stage of the disease.

A pathological vascular impairment of the choroid has already been described in diabetic patients. Interestingly, we found a CC hypoperfusion, as expressed by FDa, significantly greater in NPDR compared with NoDR group.

Several previous studies already reported a CC hypoperfusion in diabetic eyes^11,12^. Recently, a CC impairment was reported by Dai et al. in diabetic patients compared to controls by using the same metrics used in our paper (CC FD calculated with PLEX Elite SS-OCTA)^13,14^, and another recent interesting study showed microvascular changes in the retinal (SCP and DCP) and choroidal levels (CC) in both, T1D and T2D patients, compared to controls, even in the absence of DR (NoDR)^15^.

Our data agree and support this evidence: in fact, as already demonstrated, the reduction of CC flow increases with the advancing of diabetic retinopathy^12^, and we reported worsening of CC flow between NoDR and NPDR groups.

When correlating the EZ “normalized” reflectivity with CC FD parameters, we found a negative correlation between EZ “normalized” reflectivity and CC FD% in NoDR group.

Dai et al., suggested that the CC FD might represent an earlier preclinical marker of microvascular dysfunction, as demonstrated by the significantly increased in CC FD in NoDR eyes compared to controls^13^.

This result could support the hypothesis that the CC vascular dropout is associated with photoreceptors’ damage also before the development of diabetic retinopathy^16^, confirming the involvement of choroid in diabetic physio-pathological events (“diabetic choroidopathy”)^17,18^. Indeed, photoreceptors and CC have been described as elements of a symbiotic unit in which dysfunction of their components are strictly correlated also in a different degenerative pathology, like intermediate age-related macular degeneration (iAMD)^8,19^.

By contrast, not statistically significant relationship has been found between EZ “normalized” reflectivity and CC FD parameters in TD1 NPDR group.

When comparing electro-functional data between NoDR and NPDR eyes, we did not find any significant differences in all studied areas, meaning that the bioelectrical function of the pre-ganglionic elements is grossly similar either in absence or presence of minimal clinical signs of diabetic retinopathy. This agrees with all that found in our previous study^9^ by applying both ring and sector analyses to signals recorded from the macular area up to 10° of foveal eccentricity. As already described^9^, although outer retinal signals are different between controls and T1D eyes, our data confirms that mfERG is not a tool able to discriminate between different stages of the DR disorder.

Moreover, when correlating functional data with OCTA parameters, we found a significant correlation between reduced mfERG RADs and increased CC FDa and CC FD% in R3 and the combined R1 + R2 + R3 areas in NPDR eyes.

This finding suggests that only in presence of clinical evident signs of DR the photoreceptors and bipolar cells’ dysfunction correlates with the CC flow impairment, and significantly only in the parafoveal areas or in the combined foveal and parafoveal areas.

This means that for NPDR eyes, in those localized areas of the macula where a major number of retinal abnormalities are visible (micro-haemorrhages, microaneurysms, non-perfusion areas…), the underneath outer retinal elements are functionally impaired in proportion to choroidal vascular supply deficit^4^.

The above-mentioned correlation with CC features is not present in NoDR eyes with absence of microvascular retinal abnormalities and with good glycaemic control. It is likely that in the early stage of DR, the impairment of the CC flow is not associated to the precocious parafoveal retinal dysfunction.

In addition, we did not find either in NPDR or in NoDR eyes any significant correlation between mfERG data and EZ “normalized” reflectivity. This finding may be ascribed to the fact that when recording data by mfERG we mainly select the functional activity of photoreceptors and bipolar cells and their synapse, which mainly involves the outer nuclear and outer plexiform layers. It is likely that the precocious reflectivity impairment of the EZ can eventually interfere with the activation of phototransduction occurring at the level of the outer segment of photoreceptors and not with the neuronal signalling impairment involving outer and middle retinal layers, thus it could not be captured by the mfERG responses. Another hypothesis could be that, in the diabetic neurovascular disorder, the choroidal ischemia and the CC flow deficit are likely able to induce a precocious preganglionic element dysfunction, before the photoreceptors’ structural damage is evident.

In our study, exploring the correlation between CC parameters and age, we found a significant correlation between CC FDa and CC FD% with age in NPDR and CC FDa and age in NoDR group. These correlations were expected and were in line with a recent study that showed a strong negative correlation between CC FD and ageing in healthy subjects in foveal, parafoveal and perifoveal areas^20^.

Interestingly, we also found a correlation between diabetes duration and CC FDa and CC FD% in NoDR group. These correlations could represent another aspect of the pathological event series for which CC flow impairment worsens with advancing diabetic disease^12^; in fact, even if in NoDR group no ophthalmoscopic sign of DR were observed, the CC perfusion could be affected by a diabetes longer time disease. These correlations could have lost in NPDR groups, in which diabetes duration may be a part of a greater complex pathological mechanism.

As for the relationship between mfERG RAD values and descriptive demographics in NoDR and NPDR examined patients, we found a significant correlation between reduced preganglionic bioelectrical activity and increasing age in the parafoveal areas only in NPDR eyes. This finding confirms the already known concept of significant functional deterioration of photoreceptors and bipolar cells in older T1D patients, when signs of diabetic lesions are visible^9^. This correlation could not be confirmed in the very early stages of the disease, as also the overall glycaemic load and disease duration were not correlated with retinal dysfunction in all examined areas in both groups**.** Our sample consisted of well controlled metabolic patients and this aspect could be responsible of the absence of a significant correlation between retinal morpho-functional dysfunction and HbA1c.

Our study presents some limitations, which includes its cross-sectional design and the relatively small sample size. Future prospective longitudinal studies on the retinal and choroidal perfusion in diabetic eyes should help to better understand the relationship between CC perfusion and photoreceptor damage.

In conclusion in this study we aimed to explore the relationship between outer retina morphology and function and choriocapillaris changes in type 1 diabetic patients with and without signs of diabetic retinopathy. Interestingly we found in NPDR T1D eyes a significant relationship between abnormal outer retina functional responses and CC impairment and in T1D eyes without DR a significant relationship between EZ “normalized” reflectivity and CC features.

## Materials and methods

### Study participants

This observational cross-sectional study enrolled T1D eyes with non-proliferative diabetic retinopathy (NPDR) and T1D without DR (NoDR) at the Department of Ophthalmology, IRCCS-Fondazione Bietti, Rome, was approved by the Institutional Review Board of IRCCS—Fondazione Bietti, Rome (protocol n. Ret03/2016) and followed the tenets of the Declaration of Helsinki. Written informed consent was obtained from all participants.

Two experienced examiners (MP and EC) identified the eyes with NPDR based on the analysis of color fundus photographs, according to the modified ETDRS retinopathy severity scale and analyzed the B-scan OCT images to exclude the presence of edema^21^.

Patients received a complete ophthalmologic examination, which included the measurement of BCVA using ETDRS visual charts, intraocular pressure (IOP), and dilated fundus examination.

Exclusion criteria were: presence of macular edema as assessed by fundus examination and confirmed by OCT exam, previous ocular surgery or intravitreal injection therapy, any maculopathy caused by other than DR, significant lens opacity and refractive error > -6 diopters spherical equivalent (SE) or >  + 4 diopters SE. Poor quality images with a signal strength index (SSI) lower than 6 for the PLEX Elite Swept Source (SS)-OCTA or with significant motion artifacts (seen as large dark or grey lines on the enface angiograms) were also excluded.

### OCTA imaging

All patients underwent OCTA imaging using SS-OCTA PLEX Elite 9000 device (Carl Zeiss Meditec Inc., Dublin, CA, USA) with a 3 × 3 field of view area centered on the fovea^22^.

The main outcome measures were: ellipsoid zone (EZ) “normalized” reflectivity and choriocapillaris (CC) perfusion density. The CC PD was analyzed as: CC flow deficits number (FDn), CC flow deficit average area (FDa) and CC flow deficit percentage (FD%).

All measurements were performed in a circular region of interest (ROI) centered on the fovea and with a radius of 1.25 mm. This decision was made because of enface images limited lateral resolution^8^.

### Quantification of the EZ “normalized” reflectivity

The EZ “normalized” reflectivity was investigated as previously reported^3,8^. Briefly, the EZ enface image was exported after checking for a correct segmentation and then imported into Fiji ImageJ software (version 2.0.0, National Institute of Health, Bethesda, MD; available at http://rsb.info.nih.gov/ij/index.html). The mean brightness of the EZ enface image was measured. Because the structures’ brightness may depend on a variety of uncontrollable factors, thus a previously described image-processing algorithm to normalize the signal across a cohort was used^3,8,23,24^.

### Measurement of the CC perfusion

The CC enface OCTA images were segmented using a 20 µm thick slab, which was placed 29 µm under the RPE fit boundary, that corresponds to Bruch’s membrane^25^. The obtained CC images were imported in Fiji ImageJ (software version 2.0.0, National Institute of Health, Bethesda, MD; available at http://rsb.info.nih.gov/ij/index.html) and each CC image was compensated adjusting for shadowing artifacts and removing retinal vessel projection artifacts^26^ and then binarized using the Phansalkar method (with a window radius of 3 pixels)^22,27,28^. In order to quantify the CC flow, the “analyze particles” command was employed. The CC flow was finally measured using the following variables: (i) the FD number (FDn) that corresponds to the number of flow voids within the circular ROI, (ii) the FD average area (FDa) that corresponds to the average size of the flow voids and (iii) the FD percentage (FD%) that corresponds to the total percentage of flow voids within the ROI.

### Functional evaluation by multifocal electroretinogram recordings

In NoDR and NPDR eyes, mfERG was recorded according to the ISCEV standards^29^ and to our previously published method by the VERIS Clinic 4.9 (Electro-Diagnostic Imaging, San Mateo, CA, USA) system^9,30–32^.

After automatic rejection of artifacts by the software, the first order kernel response was examined to measure the function of the pre-ganglionic retinal elements (photoreceptors and bipolar cells)^29^.

The functional main outcome measure was the averaged response amplitude density (RAD, measured in nV/deg^2^) between the first negative (N1) and the first positive (P1) peaks, obtained from three concentric annular retinal regions (rings = R) centered on the fovea. Ring analysis was used to differentiate changes of the bioelectrical responses of the central foveal region with respect to the more eccentric ones in the macular area. Therefore, we analyzed the N1-P1 RADs derived from 0 to 2.5 (ring 1, R1), from 2.5 to 5 (ring 2, R2), from 5 to 10 (ring 3, R3), from 0 to 5 (R1 + R2) and from 0 to 10 (R1 + R2 + R3) degrees. To compare mfERG and OCTA results from almost superimposable areas, we referred to photoreceptor topography as in Curcio et al.^33^.

MfERGs were performed three times on the same day, for test–retest variability, in each T1D subject. The recording with the highest N1-P1 RAD was considered in the statistical analysis.

MfERG signal-to-noise ratio (SNR) was estimated following the methodology used in our previous work^9,31^ and discussed by Hood and Greenstein^34^. A SNR ≥ 3 was accepted for mfERG measurement.

### Statistical analysis

NoDR, NPDR OCTA and mfERG data followed a normal distribution based on Anderson–Darling and Kolmogorov Smirnov tests.

We used a one-way analysis of variance (ANOVA) to test whether groups’ OCTA and mfERG parameters’ means differ. We focused our attention on OCTA EZ “normalized” reflectivity, FDn, FDa and FD % to compare groups’ means.

We compared mfERG groups’ parameters: R1, R2, R3, R1 + R2, R1 + R2 + R3 N1-P1 RADs. Each mfERG parameter was linearly regressed with each OCTA parameter by means of Pearson test for NoDR and NPDR groups and pooled together. Clinical features (age, diabetes duration and HbA1c) were linearly regressed with mfERG RADs and OCTA parameters for each group.

OCTA EZ “normalized” reflectivity was linearly regressed with OCTA parameters: FDn, FDa and FD % for NoDR and NPDR groups. A p-value ≤ 0.05 was chosen as statistically significant.
